# Sea anemones extract tin associated with polyvinyl chloride pre-production pellets

**DOI:** 10.1039/d6em00110f

**Published:** 2026-03-11

**Authors:** Zoie Diana, Megan Swanson, Danielle Brown, Jessica Wang, Jessica Zhao, Nelson A. Rivera, Heileen Hsu-Kim, Daniel Rittschof

**Affiliations:** a Duke University, Division of Marine Science and Conservation, Nicholas School of the Environment, Duke University Marine Laboratory, Duke University Beaufort North Carolina 28516 USA zoie.diana@utoronto.ca; b Integrated Toxicology and Environmental Health, Nicholas School of the Environment, Duke University Durham NC 27708 USA; c Department of Ecology and Evolutionary Biology, University of Toronto Toronto Ontario M5S3B2 Canada; d Duke University, Department of Civil & Environmental Engineering 236 Wilkinson Building Durham NC 27708 USA; e Plastic Pollution Working Group, Duke University Durham NC 27708 USA

## Abstract

Marine animals consume microplastics; however, it remains unknown if plastic additives can be extracted from ingested microplastics. This research utilizes animal behavior experiments and analytical chemistry to determine if sea anemones consume plastic pre-production pellets and extract lead (Pb) and tin (Sn) additives from pellets. We compared the consumption of PVC pellets to shrimp-extract-flavored PVC pellets. The time from pellet ingestion to egestion (feeding retention time) averaged 7–10 hours and did not differ between untreated (83% of pellets consumed) and shrimp-flavored PVC pellets (100% of pellets consumed). Sequential feeding of the previously consumed pellets to new anemones rapidly decreased feeding retention time until pellets were no longer consumed. To determine if anemones could extract Pb and Sn additives, we ran additional feeding trials in which treatment anemones were offered one PVC pellet daily for 10 days and control anemones were not offered pellets. We quantified lead and tin in anemones, PVC pellets, seawater, and anemone food (*Artemia* spp.) fed to anemones using inductively coupled plasma mass spectrometry, and found that treatment anemones had significantly higher tin concentrations (0.80 ± 0.07 µg g^−1^) and similar amounts of lead (0.13 ± 0.01 µg g^−1^), compared to control anemones (0.53 ± 0.06 µg g^−1^ of tin and 0.15 ± 0.02 µg g^−1^ of lead). The increased tin concentrations in treatment anemones exceeded the amount quantified in PVC pellets, suggesting that the accumulation is attributable to other sources, at least in part. Loss of variability in tin concentrations in consumed pellets suggests that loosely associated tin may explain the observed increases in tin.

## Abstract 2

Sea anemones that consumed PVC pre-production pellets had greater concentrations of tin than control anemones.

## Graphical abstract

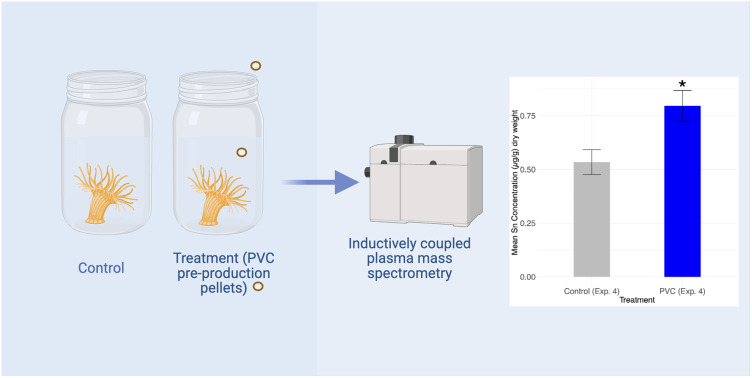

**Environmental significance**

Although many marine animals ingest plastic, the extent to which animals can accumulate plastic-associated additives remains unclear. This is important because plastic contains lead and tin additives, which impair essential biological functions like reproduction and development. Here, we use the sea anemone as a model animal and find that anemones readily consume plastic pellets and exhibit increased concentrations of tin, but not lead, as compared to control anemones. The amounts of tin in anemones exceeded those found in plastic pellets, suggesting that the tin is associated with the plastic due to manufacturing processes or ambient sources, such as seawater. The results are relevant to marine animals and ecosystems, which are increasingly facing multiple stressors like microplastics and heavy metals.

## 1. Introduction

Even with global, coordinated efforts to reduce plastic pollution, an estimated 710 million metric tons of plastic will pollute aquatic and terrestrial environments between 2016 and 2040.^1^ As of 2020, 701 species are documented to be affected by marine plastic pollution.^2^ Plastic physically harms marine animals through injury, entanglement, and, upon ingestion, gastrointestinal obstruction.^3–5^ Plastic is eaten directly, when mixed with prey,^3,6^ and unintentionally when consuming prey that has already ingested plastic. Plastic visually resembles prey^7–11^ and leaches chemicals that signal to animals that plastic is food.^12–15^

Plastic pollution poses chemical harms to marine animals due to exposure to additives, including catalysts, residual or unreacted monomers, contaminants, processing aids, and adsorbed environmental pollutants.^16–20^ Collectively, plastics contain over 16 000 chemical compounds, most of which (66%) lack data on toxicity and persistence.^18,19,21,22^ It is known that >3600 compounds from plastics include teratogens, endocrine disruptors, and carcinogens, which can cause acute toxicity and harm.^21,23–26^

Plastic producers incorporate additives into plastic during manufacturing to create durable, colorful, and malleable materials.^20,27^ Metallic additives serve as catalysts, heat and ultraviolet light stabilizers,^20,27^ colorants,^20^ biocides,^20^ and mold-release compounds.^28^ It is estimated that 2000 million metric tons of plastic additives will be produced between 2015 and 2050.^29^ Plastic additives are not bound to polymers and thus may leach into the environment.^24,27,30,31^ Plastic matrices adsorb organic and metallic pollutants from the environment^32–35^ and may deliver and remove chemicals from animals' organs and tissues after plastic consumption.

There is extensive documentation of plastic ingestion by marine animals and knowledge of metallic additives in plastic. However, it is unclear if plastic ingestion leads to the extraction and biological uptake of metallic additives. Anemones are reported to consume polyethylene pellets and extract lead (Pb) additives.^13^ Here, we pair animal behavior experiments with analytical chemistry tests to determine if metallic additives in polyvinyl chloride (PVC) preproduction pellets are bio-accessible to anemones. Our overarching research objective is to determine whether sea anemones consume polyvinyl chloride pre-production pellets (hereafter referred to as PVC pellets) and, if so, whether they can extract metal additives from pellets. We chose the sea anemone *Exaiptasia* (=*Aiptasia*) *pallida*^36^ as our test animal because it is locally abundant^37^ and hardy in the laboratory,^38^ lacks eyes, and uses tactile and chemical cues for ingestion. These anemones readily consume plastic pellets, microplastic spheres, and microfibers.^6,13,39^ Anemones also contain symbiotic zooxanthellae, which accumulate trace metals and elements.^40,41^ We focused on the metallic additives of lead (Pb) and tin (Sn) because exposure is known to have adverse health consequences and result in anemone bleaching as well as acute and chronic toxicty.^41,42^ Lastly, we expect that Pb and Sn additives may undergo transport when PVC pellets are consumed because unplasticized PVC pipes have been shown to leach tin into water when exposed to UV.^27^

## 2. Materials and methods

We conducted a preliminary test (see SI for details) and four experiments. We test two hypotheses: (1) PVC pellets stimulate feeding responses in sea anemones, and (2) anemones that consume PVC pellets will have increased concentrations of Pb and Sn, relative to control anemones. To test the first hypothesis, Experiments 1 and 2 assessed anemone PVC pellet feeding behavior to determine if anemones consumed PVC pellets and if so, quantified the time from ingestion to egestion. To test the second hypothesis, we quantified Pb and Sn in sea anemones that had consumed PVC pellets, compared with those that had not. We also quantified Pb and Sn in the laboratory system, including seawater and anemone food, to assess the relative contributions from other potential sources.

The PVC pellets used in all experiments were obtained from Raleigh Plastics (June 2017) and were stored in sealed, lidded glass canning jars in a dark room. We used the same batch of PVC pellets as in Ward *et al.* (2022).^26^

### 2.1 Anemone husbandry

Anemone husbandry was the same as Diana *et al.*, (2020).^13^ Approximately 50 *E. pallida* were collected from the floating research docks at Duke University Marine Laboratory (+34.7200, −76.6700) and were cultured in a seawater facility with air temperature 25 ± 1 °C, a 14 : 10 light: dark cycle, single-pass, sand-filtered seawater, and high-volume, low-pressure aeration through a 15 × 5 × 5 cm air stone. Anemones were cultured in rectangular (71 × 121 × 30 cm) fiberglass tanks with a standpipe-controlled depth of 24 cm. Tank temperatures were 25 ± 5 °C due to the approximately 15 mL per minute inflow of ambient seawater. Anemones were fed approximately 1 × 10^6^ newly hatched *Artemia* spp. from one teaspoon of cysts (Great Salt Lake brand) in 1 L of seawater daily. Fluorescent light strips (50 cm in length) were positioned about 15 cm above the tanks to support the anemones' photosynthetic symbionts. These lights were on at all times. In these conditions, anemones are brown due to *Symbiodinium* symbionts. Anemones grow, reproduce somatically, and spawn periodically, covering the tank's walls. Anemones for experiments were chosen from this stock.

For experiments, we haphazardly selected anemones from the culture tanks that were estimated to have an expanded tentacle crown size of approximately 2 cm. A single-edged razor blade was used to detach the anemone's pedal disk from the tank surface. Each anemone was transferred to an individual 8 cm diameter × 15.5 cm deep 1 L glass canning jar (that had been combusted at 500 °C for 4 hours) rinsed and filled with about 800 mL of 35 PSU 1 µm filtered, then aged at 25 °C seawater, hereafter referred to as seawater.^43^ Filled jars were placed on the benchtop and equilibrated to 25 ± 1 °C overnight.

### 2.2 Experiment 1: anemone feeding retention time

Individual anemones were transferred to previously combusted 1 L glass canning jars filled with approximately 800 ml of seawater and allowed to attach to the jar overnight (January 2020). Fresh anemones were offered shrimp extract-treated PVC pellets (*n* = 10) and PVC pellets (*n* = 10) using long forceps to gently drop a pellet onto the anemone's tentacle ring. The time from ingestion to egestion-feeding retention time was measured. PVC pellets were treated with shrimp extract to meet our goal of having all of the anemones consume all the pellets. Shrimp extract was generated from the tail meat of two frozen shrimp. The meat was weighed and finely minced. An equal mass of deionized water was added to make a 1 : 1 ratio of shrimp to deionized water. The mixture was homogenized using a Dounce glass homogenizer and centrifuged in a microfuge for 2 minutes at 10 000 rpm. The supernate was portioned into 100 µl aliquots and frozen at −20 °C until use.

If an anemone did not eat the first pellet offered, a second pellet was presented, and rejection or consumption of the pellet was noted. To determine if a pellet was egested, we briefly shined a UV light on the bottom of the jar. We checked the jars every fifteen minutes for 12 hours. The time that a pellet was egested was recorded.

### 2.3 Experiment 2: anemone refeeding experiment

The goal of this experiment was to determine the feeding retention times of previously consumed plastic pellets, which may provide insights on if plastic loses its flavor over repeated feedings. Anemones were transferred to combusted 1 L glass canning jars (*n* = 10) filled with filtered, then aged seawater and allowed to attach to the jar overnight. Anemones were offered one PVC pellet each. We recorded the retention time, checking for egestion every fifteen minutes. The egested pellet was retrieved and fed to a new anemone until two subsequent anemones would no longer consume the pellet.

### 2.4 Experiments 3 and 4: lead and tin uptake from PVC pellets

**Fig. 1:**
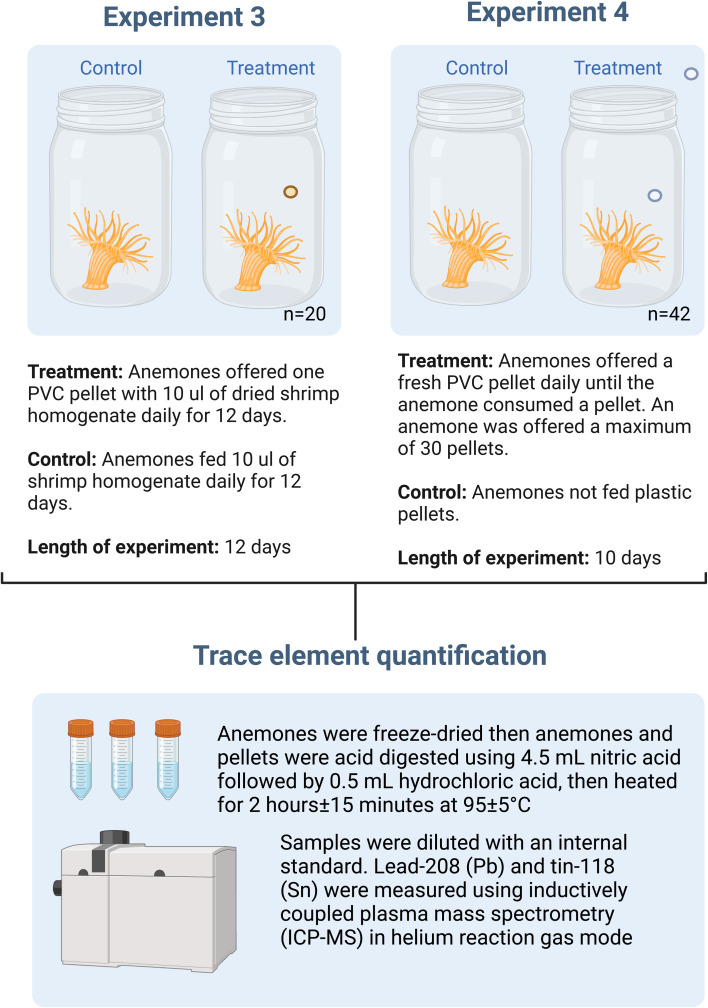
Methods summary of Experiments 3 and 4, which were designed to determine if anemones could extract lead and tin from PVC pellets. In Experiment 3, treatment anemones were offered one PVC pellet with 10 µl of dried shrimp homogenate daily while controls were offered 10 µl of the shrimp homogenate daily for twelve days. In Experiment 4, anemones were offered a PVC pellet daily for ten days until an anemone consumed a pellet, with a maximum of 30 pellets offered. Control anemones were not offered plastic pellets. The endpoint of both experiments included trace element concentration for lead and tin using ICP-MS. Figure created in BioRender.

We conducted one preliminary (see SI for the section Methods, Preliminary Test for details) and two PVC pellet feeding experiments (Experiments 3 and 4) to assess lead and tin uptake by anemones (see Fig. 1 for a methods diagram). In the preliminary experiment, anemones consumed varying amounts of pellets, which informed our feeding protocols in Experiments 3 and 4.

#### 2.4.1 Experiment 3. shrimp-extract treated PVC pellets

Because anemones consumed a variable number of untreated pellets in the preliminary experiment (see SI for details), we treated PVC pellets with 10 µl of aqueous shrimp extract in Experiment 3 (Section 2.2). Each evening, a shrimp extract aliquot was thawed to flavor PVC pellets (*n* = 10) that were fed to treatment anemones the next morning. Treatment anemones were offered one PVC pellet treated with shrimp extract daily. Consumption or rejection of the pellet was noted. Control anemones (*n* = 10) were fed 10 µl of shrimp extract daily by pipetting it near the crown of the anemones. All anemones were fed 1 mL of *Artemia* spp. daily as previously described (*N* = 20).

Anemones were photographed to count anemone tentacles over 12 days. On days 4, 8, and 12, photos (aerial perspective) of each anemone (*N* = 20) were taken at the mouth of the jar. A second photo was taken if a better angle could be accomplished. A second researcher, blinded to control and treatment group assignments, counted the number of visible anemone tentacles in each photograph (for results of tentacle counting see SI section Tentacle counting results).

On day 13, anemones were removed from jars using a razor and forceps (rinsed with seawater and then deionized [DI] water), blotted on a clean Extra Low Lint KimWipe (Kimberly-Clark), and placed in a previously weighed glass culture tube (18 × 150 mm, Fisher Scientific). Tubes containing the anemones were re-weighed. Anemones were homogenized in 2 mL of DI water using ten turns on a dounce homogenizer, reweighed, and frozen at −80 °C.

We collected seawater, shrimp extract, egested pellets (*n* = 10), and uneaten pellets (*n* = 10) to quantify Pb and Sn by ICP-MS. Five groups of unused pellets and ten pellets each from days 2, 3, 6, 9, and 12 were blotted on a KimWipe, placed in a glass culture tube, and frozen at −20 °C. Samples were transported on ice from the Duke University Marine Lab (Beaufort, NC) to Duke University (Durham, NC) and were stored at −60 °C. Anemone samples were lyophilized overnight FreeZone Plus 2.5 L Cascade Benchtop Freeze Dry System by Labconco (Kansas City, Missouri) and stored sealed at 4 °C.

#### 2.4.2 Experiment 4: anemones offered multiple PVC pellets

In Experiment 4, we doubled the number of anemones tested in order to improve resolution (*N* = 42). Control anemones were not offered PVC pellets (*n* = 20). Each evening, treatment anemones were presented with PVC pellets (*n* = 22). If an anemone did not eat the PVC pellet, a new pellet was presented until the anemone consumed a pellet. Individual treatment anemones were presented with up to 30 pellets, and the number of pellets rejected was recorded. If an anemone did not eat the pellet, we moved on to the next anemone and later returned to the anemone to offer it a fresh pellet. Rejected pellets were placed in a glass finger bowl. If pellets were egested prior to finishing the feeding trial, the egested pellets were retrieved and stored dry. The remaining egested pellets were removed the next morning and stored separately. All anemones were fed 500 µl of *Artemia* spp. each morning (*N* = 42).

On day 11, anemones were removed from the jars, blotted, weighed, and homogenized as described in Section 2.4.1. All anemones as well as uneaten, egested, and rejected PVC pellets (*n* = 10) were transported frozen from Duke University Marine Lab (Beaufort, North Carolina) to Duke University (Durham, North Carolina).

### 2.5 Trace element quantification

#### 2.5.1 Acid digestion

Anemones were freeze-dried overnight using a FreeZone Plus 2.5 L Cascade Benchtop Freeze Dry System by Labconco (Kansas City, Missouri). Acid digestions followed guidelines by the Province of British Columbia (2014)^44^ with a few modifications, described in detail in Diana *et al.*, (2020).^13^ Based on our preliminary experiment, in which anemone lead and tin concentrations fell below the method of detection, we processed at least 500 µg of anemone sample, if available, for analysis. Nine anemones (*N* = 42) had less than 500 µg mass; in those cases, all samples were used and diluted with 0.5 mL of MilliQ.

Sample digestion entailed heated acid extraction for 2 hours at 95 ± 5 °C in 4.5 mL of 67–70% nitric acid (HNO_3_) and 0.5 mL of 34–37% hydrochloric acid (HCl) mixture. After digestion, the samples were diluted with MilliQ to bring the volume back to 5 mL. The digestion series included four digestion blanks and duplicate digestion of National Institute of Standards and Technology Standard Reference Material 2976 (freeze-dried mussel tissue). All digestions were performed under a fume hood in metal-free digestion vessels with reflux caps.

Acid-digested samples were prepared for trace element quantification by diluting 250 µl of the sample with 4.82 mL of internal standard (Ultratrace 2% HNO_3_ (v/v), 0.5% HCl (v/v), and 20 ng mL^−1^ In, Rh, Te, and Ir as internal standards). Lead-208 and Tin-118 were measured on an Agilent Technologies 7900 ICP-MS operated in helium reaction gas mode. Instrument calibrations were performed and verified by running an ICP Trace Metals in Drinking Water Standard (CRM-TMDW-A Trace Metals in Drinking Water Standard A) in duplicate every 30 samples and at the beginning and end of the run. Trace elements were measured in triplicate for each sample, averaged, and reported as µg per g (dry weight [d.w.]). We calculated the low point on the calibration curve, method detection level (MDL), and practical quantitation limit (PQL) based on instrument detection limits for elements. MDL is the minimum concentration reported with 99% confidence that the sample analyzed can be distinguished from the blank based on instrumental limitations for Pb and Sn (0.05 µg L^−1^ of Sn and 0.01 µg L^−1^ of Pb).^45^ PQL is based on the lowest point on the calibration curve for each ICP-MS run.

### 2.6 Data analysis

Unless stated otherwise, R and JMP were used to create figures and conduct statistical analyses. Data were checked for normality using a Shapiro–Wilks test. When the data were normally distributed, an ANOVA was used, followed by a Tukey's post hoc test. If data were not normally distributed, a Mann–Whitney *U* test was used to compare differences between treatment groups. Statistical significance was *p* < 0.05. Throughout the results, bars in figures show the mean values, and error bars show the standard error of the mean (SEM).

## 3. Results

### 3.1 Anemone feeding retention time

**Fig. 2:**
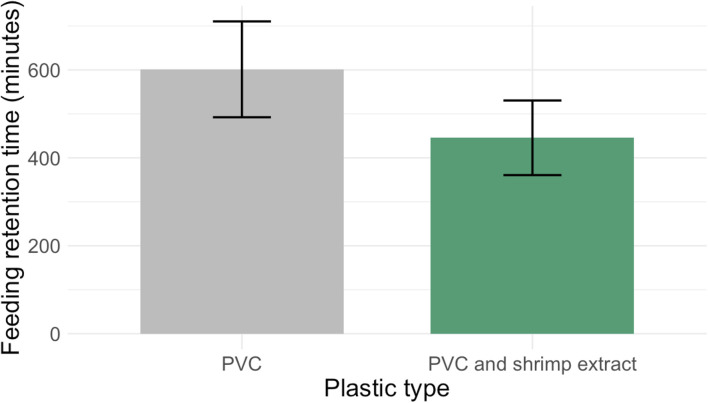
Anemone feeding retention times are similar between untreated PVC pellets and shrimp extract-treated PVC pellets. Anemones retained PVC pellets for 601.3 ± 109 minutes and shrimp extract-treated PVC pellets for 445.6 ± 84.9 minutes (*p* > 0.05, Mann–Whitney *U* test) (*N* = 20 anemones total).

Six of ten anemones (*n* = 10) consumed one unflavored PVC pellet. All anemones (*n* = 10) consumed one shrimp-extract-treated PVC pellet. Anemones retained PVC pellets for 601.3 ± 109 minutes, and shrimp treated PVC pellets for 445.6 ± 84.9 minutes (see Fig. 2 for mean feeding retention times). Feeding retention times were not normally distributed (Shapiro–Wilks, *p* < 0.05) and were similar between the PVC and shrimp extract-treated PVC pellets (Mann–Whitney *U* test, *p* = 0.70). When we stopped measuring feeding retention times at twelve hours, four anemones had not egested untreated PVC pellets, and three anemones had not egested shrimp treated PVC pellets.

### 3.2 Experiment 2: anemone refeeding experiment

**Fig. 3:**
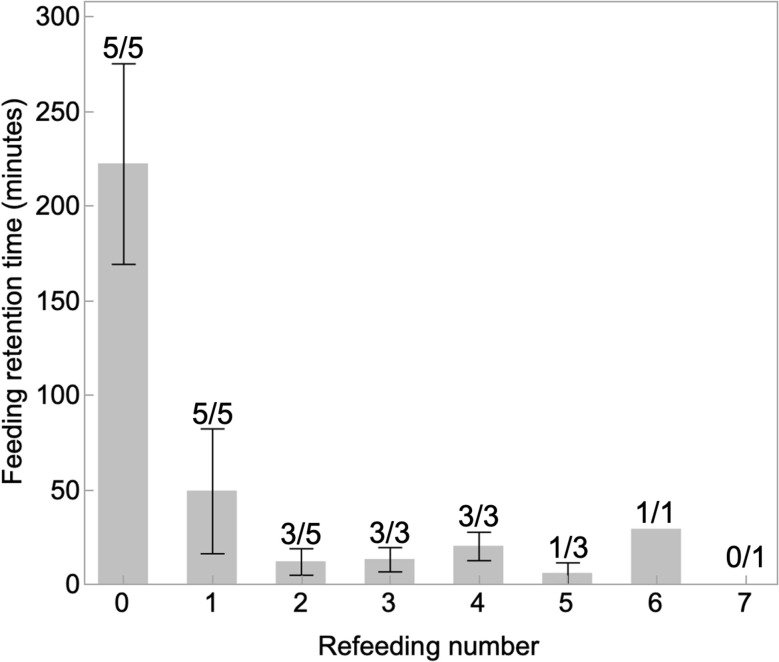
Decreasing feeding retention time with sequential presentation of previously consumed PVC pellets to new anemones. Numbers over the bars show the number of pellets consumed out of the total offered (*e.g.*, for feeding 0, five pellets were consumed out of five total offered [5/5]). The initial feeding is indicated by a zero, and subsequent feedings are noted. The retention time for the first refeeding was fourfold less than the initial feeding. Anemones stopped ingesting pellets with each subsequent presentation until no pellets were consumed by the seventh refeeding.

Anemone refeeding retention times were normally distributed for most of the refeedings (Shapiro–Wilks, *p* > 0.05). Feeding retention times decreased over repeated feedings of the same pellet to fresh anemones (Fig. 3, one-way ANOVA, *p* < 0.05) with feeding 0 and 1 significantly different from feedings 2, 3, 4, and 5; the other feedings were not significantly different from one another (Tukey's posthoc). Of the 5 pellets tested, all 5 were eaten initially (feeding 0) and then consumed at the first refeeding (feeding 1). At the next refeeding, two pellets were not eaten each by two anemones and three pellets were eaten (feeding 3). The three pellets were each eaten and then rapidly egested for feedings 3 and 4. One pellet was eaten at feedings 5 and 6, and not at feeding 7.

### 3.3 Experiments 3 and 4: anemone PVC pellet consumption

The number of pellets consumed by anemones and the concentrations of lead and tin by dry weight of anemones (Section 3.3.1) are reported. All anemones consumed multiple plastic pellets (see Table 1 for a summary of the number of pellets offered and consumed). In the preliminary experiment, each treatment anemone consumed a range of 4 to 11 pellets (on average, 7.9 ± 0.6) of the twelve pellets offered. In Experiment 3, each treatment anemone consumed 12 pellets (1 pellet daily for 12 days) (*n* = 10). In Experiment 4, treatment anemones consumed a range of 3 to 10 pellets (on average 8.3 ± 0.4 pellets) (*n* = 22). On average, anemones consumed either the first or second pellet offered (mean of 1.8) of the up to 30 pellets offered daily.

**Table 1:** Summary of anemone consumption of PVC pellets. The number of PVC pellets consumed by treatment anemones in the preliminary experiment as well as Experiments 3 and 4 are shown. We report the mean number of pellets ± SEM. We also report the range of pellets consumed (minimum number to maximum number for individual anemones) as well as the range of pellets presented to anemones over the study period. The total percentage of pellets consumed by treatment anemones is shown

Experiment	Mean number of pellets consumed ± SEM	Range of pellets consumed	Number or range of pellets presented per anemone over study period	Percentage of pellets consumed
Preliminary experiment	7.9 ± 0.6	4–11	12	66%
Experiment 3 (PVC pellets + shrimp extract)	12	12	12	100%
Experiment 4 (PVC pellets offered until consumed)	8.3 ± 0.4	3–10	10–300	83%

#### 3.3.1 Experiments 3 and 4: lead and tin quantification by anemone dry weight

**Fig. 4:**
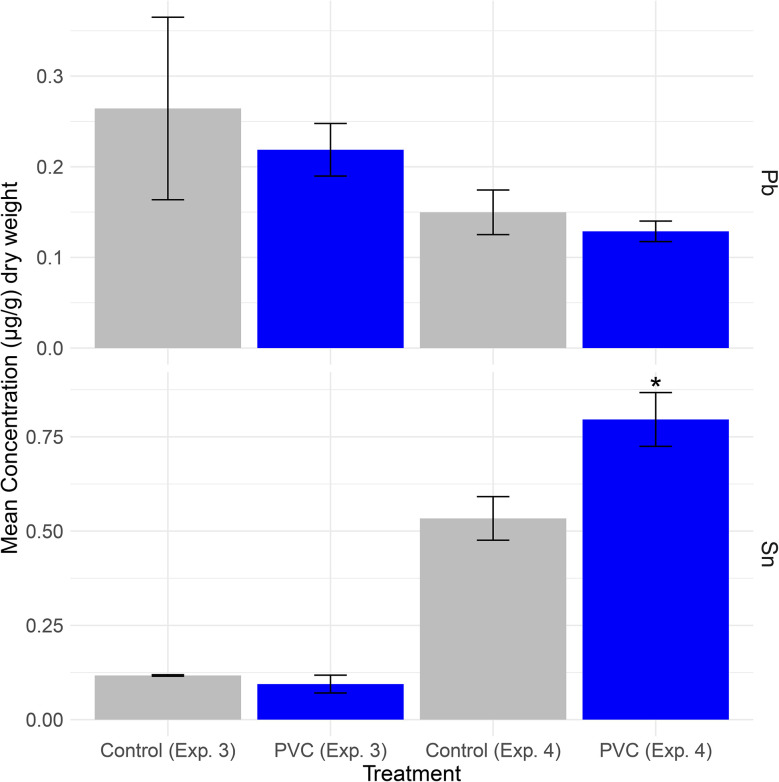
Lead and tin concentrations in control and PVC-fed sea anemones (by µg per g dry weight) in Experiments 3 and 4. The top panel shows the mean concentration of Pb in treatment and control sea anemones from Experiments 3 and 4. The bottom panel shows the mean concentrations of Sn in treatment and control sea anemones from Experiments 3 and 4. The * indicates significantly higher tin concentrations in sea anemones fed PVC as compared to controls in Experiment 4 (*p* = 0.0188, Mann–Whitney *U*).

For all samples, element concentrations that fell below the method detection limit (MDL) were excluded. In Experiment 3, all anemone Pb concentrations fell above the MDL (*N* = 20). For Pb measurements in Experiment 3, all samples fell above the PQL and MDL (*N* = 20). In Experiment 3, control anemones had a mean concentration of 0.26 ± 0.1 µg per g d.w. of Pb (*n* = 10), and treatment anemones that consumed PVC had a mean concentration 0.22 ± 0.03 µg per g d.w. of Pb (*n* = 10). In Experiment 3, control anemones and treatment anemones had similar concentrations of Pb and Sn (Mann Whitney *U*, *p* > 0.05, Fig. 4).

In Experiment 4, all anemone Pb and Sn concentrations fell above the PQL and MDL for Pb and Sn (*N* = 42). Control anemones and treatment anemones fed PVC had similar mean concentrations of Pb: 0.15 ± 0.02 d.w. µg per g for controls and 0.13 ± 0.01 µg per g d.w. for PVC-fed anemones (Mann Whitney *U*, *p* > 0.05, Fig. 4). Anemones fed PVC had significantly higher mean concentrations of Sn, 0.80 ± 0.07 µg per g d.w. (*n* = 22), as compared to control anemones 0.53 ± 0.06 µg per g d.w. (*n* = 20) (Mann–Whitney *U*, *p* = 0.0188, Fig. 4).

**Fig. 5:**
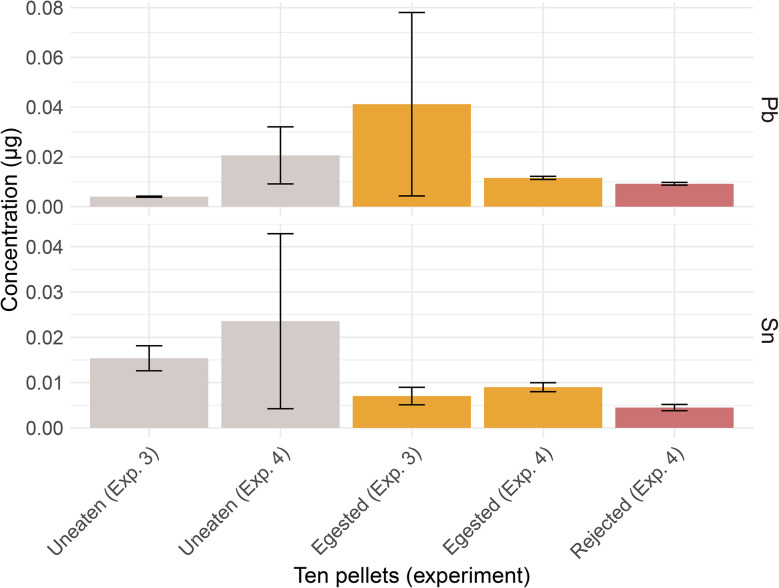
Lead and tin concentrations in ten PVC pellets that were uneaten, egested, or rejected by anemones. Ten pellets (uneaten, egested, and rejected) for Experiments 3 and 4 had similar Pb (*p* = 0.35) (top panel) and Sn concentrations (bottom panel) (*p* = 0.086, Kruskal–Wallis).

Uneaten and egested pellets from Experiments 3 and 4 had similar amounts of Pb and Sn (Fig. 5). By experiment, PVC pellets (uneaten, egested, rejected) had similar Pb (*p* = 0.35) and Sn concentrations (*p* = 0.086, Kruskal–Wallis). Uneaten pellets had a mean concentration of 0.0040 ± 0.0002 µg of Pb in Experiment 3 and 0.021 ± 0.01 µg of Pb in Experiment 4 (*n* = 10). Egested pellets had a mean concentration of 0.041 ± 0.04 µg of Pb in Experiment 3 and 0.012 ± 0.0006 µg of Pb in Experiment 4 (*n* = 10). Uneaten pellets had a mean concentration of 0.015 ± 0.003 µg of Sn in Experiment 3 and 0.024 ± 0.03 µg of Sn in Experiment 4 (*n* = 10). Egested pellets had a mean concentration of 0.0070 ± 0.002 µg of Sn in Experiment 3 and 0.0090 ± 0.001 µg of Sn in Experiment 4 (*n* = 10). Rejected pellets (Experiment 4 only) had a mean concentration of 0.0045 ± 0.0007 µg of Sn (*n* = 10). For Pb and Sn concentrations by gram of PVC, see Table S1 (SI).

Mean Pb and Sn concentrations in seawater, *Artemia* spp., and shrimp extract from Experiments 3 and 4 that were above the MDL are presented in Table 2. Because 1 mL samples of seawater approached detection limits for Experiment 3, 2 mL of seawater and *Artemia* spp. were used in Experiment 4 (see detailed methods in Section 2.4). For Experiment 3, one shrimp extract sample fell below the MDL and two fell below the MDL for Pb. In Experiment 3, two seawater samples fell below the MDL for Pb and Sn and one additional sample fell below the MDL for Pb only. Three of five samples fell below the MDL for Sn for seawater from Experiment 4.

**Table 2:** Shrimp extract, seawater, and *Artemia* spp. Pb and Sn concentrations. The mean concentrations of Pb and Sn and the SEM are shown for shrimp, extract, seawater, and *Artemia* spp

Sample	Experiment	Mean Pb (ug g^−1^) ± SEM	Mean Sn (ug g^−1^) ± SEM
Shrimp extract (*n* = 5)	3	0.00233 ± 0.00091	0.0109 ± 0.0037
Seawater (*n* = 4)	3	0.000568	0.0665 ± 0.063
Seawater (*n* = 2)	4	15 ± 8.9	2.72 ± 1.7
*Artemia* spp. (*n* = 3)	4	3.82 ± 0.022	0.739 ± 0.01

## 4. Discussion

This study examined anemone feeding behavior using PVC pellets and determined if PVC consumption impacted anemone Pb and Sn concentrations. Sea anemones consume plastic pre-production pellets at varying rates across studies and plastic types tested. Anemones consumed 66–83% of the unflavored PVC pellets offered (Table 1). In our previous study, anemones were offered National Institute of Standards and Technology pre-production PE pellets for 12 days and consumed 90.8%, 85.8%, and 84.3% of low-density polyethylene (LDPE), high-density polyethylene II (HDPE II), and high-density polyethylene III (HDPE III), respectively.^13^

In contrast to PE and PVC pellet feeding rates, only 25% of anemones consumed nylon microfibers,^39^ suggesting that PE and PVC elicit a greater feeding response than nylon microfibers. Factors that may play a role include the plastic flavor through leachate as well as the morphology and size of the particles. Another species of sea anemone, *Actinia equina*, consumed 20% of food-grade alkathene pellets offered and 13% of biofilmed pellets.^46^ These results suggest that polyethylene seems most like food to sea anemones, followed by PVC, nylon microfibers, and then alkathene. When comparing plastic pellets, which are similar in morphology, we expect that the polymer and chemical additives contribute to variable anemone feeding behaviors, with some polymers, such as PE, more closely resembling food than others.

Similar to our study, others have also found that anemones readily consume plastic flavored with prey.^6,46^ Okubo *et al.*, (2018) found that anemones consumed 3, 6, and 11 µm polystyrene (PS) microspheres mixed in *Artemia* spp.^6^ Davenport *et al.*, (2011) found that *A. equina* consumed 10 of 15 (67%) alkathene pellets coated in mussel extract.^46^

Anemones feed when triggered by physical and chemical cues.^47,48^ Cnidocytes fire and attach to the surface of plastic pellets.^12^ The shape of preproduction pellets is somewhat similar between plastic types; however, the densities, polymers and additive chemistries vary between PVC, alkathene, LDPE, HDPE II, and HDPE III. Crude oil is known to stimulate feeding in *Cnidaria*^12,49^ and residual contamination from the production process may be a contributor to anemone feeding behavior here. Since 99% of plastics are fossil fuel products,^50–52^ contaminants at the surface of plastic pellets may stimulate feeding.^17,18^ Given that different plastics each contain hundreds to thousands of compounds^16,18–20,24^ and chemical mixtures are potent phagostimulants for marine invertebrates,^53,54^ varying mixtures of hydrocarbon contaminants could result in variable plastic ingestion rates. Thus, we suggest that plastic chemistries have different flavors and different rates of leaching, contributing to differing consumption.

Evidence suggests that anemones will not consume any material offered, lending to our suggestion that plastic chemistry triggers feeding. For instance, Thorington and Hessinger (1988) found that only one anemone out of 30 had a slight response (determined by tentacle adherence as a proxy for cnidae discharge) to gelatin or agarose pellets but did not respond to glass rods.^47,48^ Anemones exhibited responses to many proteins, glycoproteins, and mucins (*e.g.,* α-casein, cytochrome-C [horse], pepsin [porcine], trypsin, and hemoglobin) and strong responses to pellets and glass rods with egg white, myoglobin [equine], ovalbumin [hen], polylysine, α-globulin [bovine], serum albumin, submaxillary mucin, and gastric mucin.^48^ Anemones also responded to low molecular weight amino acids and lipids.^48^ We have found that *E. pallida* consumes muffled glass fiber filters but retains them for a shorter period than plastic (unpublished data). Given that plastic does not contain proteins, amino acids, and nucleotides, mixtures of hydrocarbon compounds and metals on the surface of the pellets contribute to the flavor of plastic.

Anemones consume plastic in the environment. For example, the sea anemone *Bunodactis reynaudi* consumed flexible packaging (greater than 5 mm in size) at False Bay, South Africa.^55^ In the Bay of Biscay, Spain, *A. equina* consumed primarily PE, sometimes containing adsorbed compounds.^56^ Notably, *A. equina* is consumed by humans as a delicacy or *via* supplements, potentially resulting in food security and human health risks.^56^ In the lab and the environment, sea anemones consume a range of plastics at varying rates.

Refeeding experiments showed that anemones decreased the feeding retention time of PVC pellets after one refeeding, showing a similar steep decline in feeding retention time as PE pellets.^57^ A simple explanation for this decrease in feeding retention time is that the digestive process removes leachates from the pellet surface, thereby reducing the flavor that elicits feeding responses in subsequent anemones. We also noticed that the pellets developed a brown hue over the 5-year time frame, despite being stored in a rarely used, dark, windowless room in tightly lidded containers. Oxidation may have accelerated the rate of release, leading to a rapid loss of flavor. Future research could photograph the plastic pellets during a study and categorize the color using a Yellowness Index (ASTM *D* 1925–70 or *E* 313–15 × 10^1^).^17^

In a previous study, we found that anemones that consumed HDPE III pellets daily for 12 days had higher Pb concentrations than control anemones that did not consume plastic.^13^ The amount of Pb found in anemones was about 10% of that found in PE pellets Diana *et al.*, (2020).^57^ In an avian physiological model, Turner (2018) and Turner and Lau (2016) demonstrate that beached polyurethane can be a source of Pb, with about 10% of all Pb in the plastic being available for extraction.^58,59^ In this study, treatment anemones that consumed PVC had higher tin concentrations than controls. However, the increase in tin concentration in treatment anemones compared with controls was approximately 0.27 µg g^−1^ on average, which was higher than the average of ten uneaten PVC pellets (0.072 ± 0.06 µg g^−1^ of Sn). The increased concentrations of Sn in treatment anemones as compared to controls were over triple that available from the pellets alone, though Sn amounts were highly variable between pellets. It is possible that anemones elicited a greater feeding response to pellets with greater Sn concentrations than others, resulting in high concentrations. Seawater and *Artemia* spp. in Experiment 4 (see Table 2) also contained Sn that may result in increased concentrations of Sn; however, control anemones were exposed to the same seawater and *Artemia* spp. as treatment anemones. It is possible that Sn from the seawater or *Artemia* adsorbed to the PVC pellets offered to the treatment anemones and resulting in increased Sn concentrations beyond what was available just in the pellet, given that plastic litter has been shown to adsorb metals from the environment.^32,60–62^ The greatly reduced variance and lower levels of tin post exposure suggest that the pellets surfaces had loosely associated tin.

Our ICP-MS data indicate that there is greater variance in tin concentrations in uneaten pellets compared to pellets consumed by sea anemones. This suggests that loosely associated tin on the surface of the pellets during processing may also be a source of the elevated tin levels in anemones fed PVC. An experimental treatment in which PVC pellets are exposed to seawater and seawater with *Artemia* spp. without anemones would aid in differentiating if Sn from PVC processing or Sn from the seawater or *Artemia* spp. contributed to the increased concentrations of Sn observed in anemones that consumed PVC pellets, as compared to controls. In addition, further developments in standard reference materials for plastics with known quantities of additives are needed.^63,64^ Currently, the additives in plastics are not standardized across or within polymers,^18–20,22^ which makes it difficult to study additive extraction and leaching and compare results across experiments and studies.

Understanding whether plastic can transport or be a source of Sn and other contaminants in the environment is important for the health of marine animals broadly. This study measured tin-118, a stable isotope of tin used as a proxy for total tin. However, further research should examine other chemical speciation of tin, as inorganic tin species have low solubility in water while organotin compounds are environmentally-relevant and important.^65^ For example, organotins bleach sea anemones,^66^ disrupt endocrine systems of marine mollusks,^67^ and result in acute and chronic toxicity.^42^ Although some trace elements are essential for biological processes, other elements like Pb and Sn can be toxic and induce oxidative stress depending on the metal and concentration.^41^ Future research should utilize cellular and molecular markers to determine if stress is occurring and chronic exposures are also needed to better understand health effects and inform risk assessment.^68^ Research continues to report that marine animals are consuming plastic and extracting additives. Further reports of the physiological effects of plastic consumption are needed understand the effects on marine animal health.

## Conclusions

This research shows that anemones readily consume PVC pellets and that feeding retention time declines with repeated feedings of the same pellets to new anemones. The plastic additives in the pellets do not appear to be the sole source of lead or tin to the sea anemones. This work suggests that plastics may transport or contribute to the accumulation of tin found in marine ecosystems, such as seawater or organisms. Our research supports the hypothesis that plastic additives are flavors and extracted by animals upon plastic consumption. Future research could examine if plastic consumption results in adverse health consequences for sea anemones. This research provides evidence that anemones consume PVC readily and that PVC may transport metals in a marine setting.

## Author contributions

Conceptualization – Z. D., D. R. data curation – Z. D., N. R. formal analysis – Z. D. funding acquisition – Z. D., D. R. investigation – Z. D., N. R., M. S., D. B., J. W., J. Z. methodology – Z. D., D. R., N. R., H. H. K. Project administration – Z. D. resources – D. R., H. H. K. software – D. R., H. H. K. supervision – D. R., H. H. K., N. R. validation – Z. D., D. R., H. H. K., N. R. visualization – Z. D. writing – original draft – Z. D. writing – review & editing – Z. D., D. R., H. H. K., N. R., M. S., D. B., J. W., J. Z.

## Conflicts of interest

There are no conflicts of interest to declare.

## Supplementary Material

EM-028-D6EM00110F-s001

## Data Availability

All data is shared in the article and supplementary information (SI). Supplementary information: details on the preliminary test, tentacle counting, and data on the Pb and Sn in PVC pellets. See DOI: https://doi.org/10.1039/d6em00110f.
