# Supplementary material for: Sea anemones extract tin associated with polyvinyl chloride pre-production pellets
Source: Environ Sci Process Impacts. 2026 Mar 11;28(5):1233–42. doi: 10.1039/d6em00110f (PMC12998434; doi:10.1039/d6em00110f)
Supplement: EM-028-D6EM00110F-s001 [file EM-028-D6EM00110F-s001.pdf]

This journal is © The Royal Society of Chemistry 2026

Supplementary Information

Preliminary Test

Methods

In the preliminary test, treatment anemones were offered one PVC pellet daily for 12 days and control anemones were not offered plastic pellets following the same feeding behavior protocol in Experiments 3 and 4 (see **Sections 2.4.1** and **2.4.2**) as well as the trace element quantification methods (see **Section 2.5**). Because anemones consumed at minimum 4 pellets and a maximum of 11 pellets (in total 66% of pellets offered) (see Table 1 for a full summary of the results), we changed our anemone feeding protocol for experiments 3 and 4.

Results

In the Preliminary Experiment, only two control anemones and five treatment anemones fell above the MDL for Sn (N = 20 anemones total). Control anemones had a mean concentration of 0.12± 0.002 μg/g of Sn. The treatment anemones had a mean concentration 0.094± 0.023 μg/g of Sn. All anemones fell above the MDL for Pb in the preliminary test. Control anemones had a mean concentration of 0.26± 0.10 μg/g of Pb and treatment anemones had a mean concentration 0.22± 0.03 μg/g of Pb. The Preliminary Experiment results informed our decisions to alter the feeding behavior methods in Experiment 3 and increase the sample size in Experiment 4.

Tentacle counting results

Anemone tentacles were similar between control and treatment groups across the study (repeated measures ANOVA, $p>0.05$) (see **Figure S1** for the mean number of tentacles

on days 4, 8, and 12 and **Figure S2** for randomly-chosen photographs used for tentacle counting). The mean number of tentacles for control anemones on days 4, 8, and 12 was 26 ± 1.5, 26.2 ± 1.8, and 24.7 ± 2.0, respectively. For the treatment anemones fed PVC, the mean number of tentacles on days 4, 8, and 12 was 26.4 ± 2.5, 25.1 ± 1.6, and 22.6 ± 1.7, respectively.

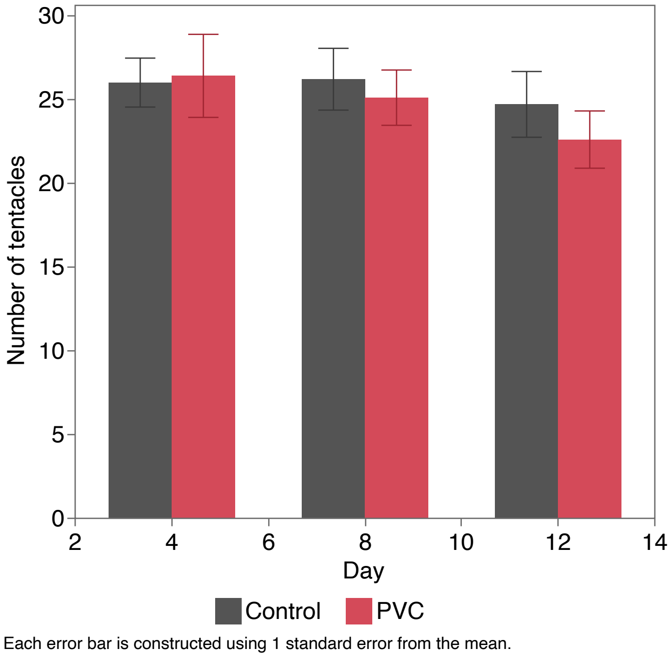

Supplemental Figure S1. Number of anemone tentacles is similar across days and treatment.

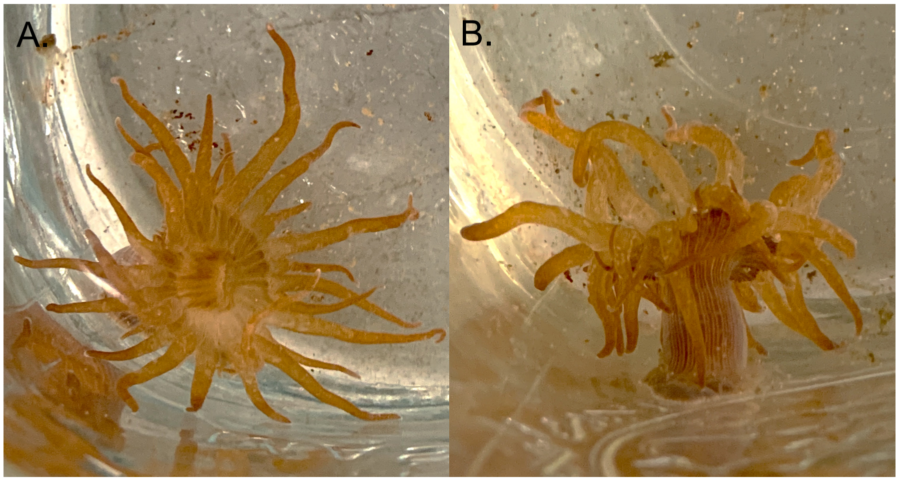

Figure S2. Randomly chosen photographs of control and treatment anemones used for tentacle counting. A random number generator was used to choose the example photographs shown here. The photograph of the control anemone was taken on day 4 and the photograph of the treatment anemone is from day 12. Photographs were brightened for clarity.

Table S1. Pb and Sn quantities in uneaten, egested, and rejected PVC pellets (Experiments 4 and 5).

Samples	Pb (µg/kg) ± SEM	Sn (µg/kg) ± SEM
Uneaten PVC pellets (Experiment 3)	11.751±0.603	44.564±7.581
Egested PVC pellets (Experiment 3)	120.972±108.326	20.747±5.483
Uneaten PVC pellets (Experiment 4)	61.640±34.497	71.631±59.093
Egested PVC pellets (Experiment 4)	33.400±1.547	25.984±2.881
Rejected PVC pellets (Experiment 4)	26.620±1.587	12.996±2.020
